# HLA Polymorphisms and Haplotype Diversity in Transylvania, Romania

**DOI:** 10.1155/2019/1342762

**Published:** 2019-12-30

**Authors:** Mihaela L. Vică, Horea V. Matei, Cosmina I. Bondor, Gheorghe Z. Nicula, Costel V. Siserman, Luminița Loga, Lucia Dican

**Affiliations:** ^1^Department of Cell and Molecular Biology, “Iuliu Haţieganu” University of Medicine and Pharmacy, 6 Pasteur Street, 400 349 Cluj-Napoca, Romania; ^2^Legal Medicine Institute Cluj-Napoca, 3-5 Clinicilor Street, 400 006 Cluj-Napoca, Romania; ^3^Department of Medical Informatics and Biostatistics, “Iuliu Haţieganu” University of Medicine and Pharmacy, 6 Pasteur Street, 400 349 Cluj-Napoca, Romania; ^4^Department of Legal Medicine, ‘Iuliu Haţieganu' University of Medicine and Pharmacy, 3-5 Clinicilor Street, 400 006 Cluj-Napoca, Romania; ^5^Clinical Institute of Urology and Renal Transplant, 2 Clinicilor Street, 400 000 Cluj-Napoca, Romania; ^6^Department of Biochemistry, “Iuliu Haţieganu” University of Medicine and Pharmacy, 6 Pasteur Street, 400 349 Cluj-Napoca, Romania

## Abstract

Transylvania is a historical region in the northwestern part of Romanian with a rather heterogeneous population. Our study is the first to determine human leukocyte antigen (HLA) profiles in a large population sample from this region and to compare them with other European population groups. HLA genes were examined in 2,794 individuals using the Single Specific Primer-Polymerase Chain Reaction (SSP-PCR) and Polymerase Chain Reaction Sequence-Specific Oligonucleotide (PCR-SSO) methods. All samples were tested for the HLA-A locus, 2,773 for HLA-B, 1,847 for HLA-C, and 2,719 for HLA-DRB1 loci. HLA gene frequency data from several European population groups (as presented in studies involving more than 1,000 individuals) served as reference in comparison with the local sample. The distribution of HLA genes in the studied population group was heterogeneous, as the Hardy-Weinberg equilibrium was statistically significant (*P* value < 0.01). The most common genes found in our sample group were A^∗^02 (0.27%), B^∗^35 (0.14%), C^∗^07 (0.25%), and DRB1^∗^11 (0.19%). The most common haplotype was A^∗^01~B^∗^08~C^∗^07~DRB1^∗^03 (1.26% in 1,770 individuals with complete data). This analysis confirmed the known heterogeneity of the Transylvanian population. The study indicates that the European population groups located in close vicinity (those from Serbia, Hungary, Wallachia, and Croatia) are genetically closest to the Transylvanian population.

## 1. Introduction

The major histocompatibility complex (MHC) is a large gene complex (approximately 3.5 million base pairs) with an integral role in the immune system. Known as the human leukocyte antigen (HLA), the human MHC-encoded glycoproteins are vital in the body's immune defence, being specialized in the presentation of short peptides to T cells [1].

The 4 Mb region of the human chromosome 6p21 designated as the MHC is the most dense and polymorphic one in the human genome [2]. The extensive polymorphism at the HLA loci reflects the importance of the encoded molecules in human transplantation and autoimmunity, as well as in regard to drug response and susceptibility to infection [3]. Compatibility in the HLA system is vital in hematopoietic stem cell transplantation [4].

The distribution of the HLA genes is a particularity of any given ethnic group. Functional differences at the HLA loci observed between related populations, better explained by changes in the frequency of an existing allele than by migration history, contribute to a divergent immune response. Genetic drifts are generally eliminated through selection processes [5].

Analysis of the HLA profiles in various ethnic groups is important since a significant number of human diseases are more common among individuals carrying certain HLA genes. HLA disparity in mating partners provides a better genetic baggage for eventual offspring. The HLA genes may influence the human lifespan, but such association rests both on the genetic background and environmental influences [6].

Transylvania (“terra ultra silvam” in Latin, i.e., the land beyond the forest) is a historical region of 100,293 km^2^ (42% of the Romanian surface), geographically located within the Carpathian arch. It neighbours Ukraine in the north, Hungary in the west, and Serbia in the southwest. Across the Carpathian Mountains lie Walachia (south) and Moldavia (east), the other two major Romanian provinces [7–9].

The territory of Transylvania has been inhabited since pre-Christianity by the Dacians, part of the Thracian population formed around 2000 BC from the blending of the Indo-European migratory populations with the native Neolithic population [7, 8, 10]. The Romans conquered Dacia in 106 AD and administered it until 271 AD, an intense process of Roman colonization taking place during that period [7, 8, 10]. In the seven centuries following the withdrawal of the Roman administration, the intracarpathic territory was successively invaded by Visigoths, Huns, Gepids, Avars, Slavs, Bulgars, Magyars, and Pechenegs [7, 8, 10]. As a result, some local population groups found shelter into the mountainous areas where they have been surviving through centuries, somewhat isolated from the rest of the population [7].

In contrast to the neighbouring territories, the massive early Slav populations migrating in the 6th and 7th centuries were virtually assimilated here by the Dacian-Roman natives [8]. However, the historical evolution of the province was significantly marked by the establishment of the Magyars and the Szeklers in Pannonia in the 9th century, their gradual advance eastwards into the territory of Transylvania paralleling an extensive process of colonization with Saxons in the 11th-13th centuries. Several successive waves of Roma populations also settled here following the Mongol invasion in 1241 [7, 8, 10].

Transylvania became an independent principality under the sovereignty of the Ottoman Empire following the occupation of the Hungarian Kingdom by the Turks (1526) and, later on, an autonomous principality part of the Habsburg Empire (1699-1867, during which time the colonization of the southwestern Transylvania region with Swabians of German origin was observed). From 1867 to 1918, it was incorporated into the Austro-Hungarian Empire under the Hungarian crown. Finally, in 1918, Transylvania became an integral part of Romania [7, 8]. All this complex historical evolution of Transylvania explains the multiethnic character of the local population.

According to the 2011 census, the total population of Transylvania was about 6.7 million inhabitants, of which 70.62% were Romanians. Minorities included Hungarians (17.92%), Romani (3.99%), Ukrainians (0.63%), Germans (0.49%), and other ethnic groups (0.77%) [9].

Our study is aimed at determining the HLA profiles (HLA-A, HLA-B, HLA-C, and HLA-DRB1) in a Transylvanian population group. This is the first attempt to analyze the frequencies of these HLA genes in a large population sample from this region.

## 2. Materials and Methods

### 2.1. Subjects

**Figure 1:**
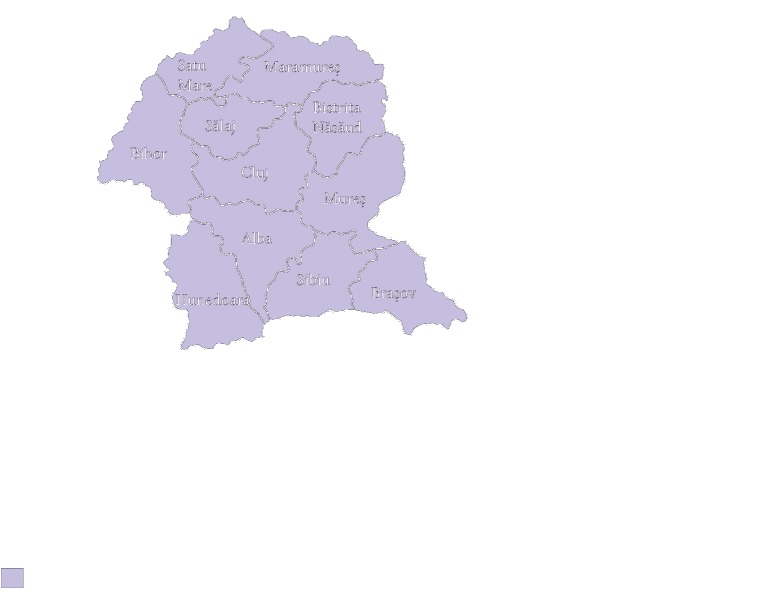


A total of 2,794 individuals of Transylvanian origin were enrolled in the study between January 2010 and December 2017. 2,262 of them were recruited from the participants in the Romanian Bone Marrow Volunteer Donors Program registered in the Romanian Bone Marrow Donor Registry by the Institute of Urology and Renal Transplant in Cluj-Napoca. Related persons were excluded based on Registry evidence. 532 unrelated individuals (mothers and presumptive fathers) subjected to paternity testing at the Institute of Forensic Medicine in Cluj-Napoca were included in the study to improve the chances to detect low frequency genes.  Figure 1 highlights the counties of origin for the Transylvanian population sample. Informed consent was obtained from all individual subjects included in the study. The study was approved by the ethics committees of both the University of Medicine and Pharmacy in Cluj-Napoca (no. 272/16.06.2017) and the Renal Transplantation in Cluj-Napoca (no. 2392/06.12.2018).

Data regarding HLA gene frequencies in several European countries neighboring Transylvania or various historically linked regions served as reference in comparisons with the local population group providing that they were the result of population studies involving more than 1,000 individuals (Table 1). Data were extracted from the Allele Frequency Net Database (http://www.allelefrequencies.net) [11], as well as from studies on the population groups of Walachia [12], Hungary [13], Serbia [14], Croatia [15], and Italy [16]. As only a few of these studies concerned haplotype analysis, we took into consideration additional data from other studies, regarding population groups in Germany [17], Bulgaria [18, 19], Macedonia [20], and Greece [21].

### 2.2. DNA Extraction

Two ml of peripheral venous blood was collected from each person subjected to paternity testing, and DNA was extracted using a Ready DNA Spin Kit (Inno-Train Diagnostik GmbH, Kronberg, Germany) according to the manufacturer's instructions. DNA concentration and purity were quantified by nanophotometric readings against a reference Tris buffer. When the value of the A260/280 absorbance ratio was outside the 1.6-2.0 range, the DNA was purified using an Epicentre MasterPure™ Complete DNA and RNA Purification Kit (Illumina Company, Madison, WI, USA) according to the manufacturer's instructions.

The same amount of blood was collected from the volunteers included in the Bone Marrow Donors National Program, DNA being extracted from the whole blood sample using an innuPREP Blood DNA Mini kit IPC16 (Analytik Jena AG, Germany) according to the manufacturer's instructions. DNA concentration was adjusted to 30 ng/*μ*L.

### 2.3. HLA Typing

The HLA-A, HLA-B, HLA-C, and HLA-DRB1 genes were typed making use of several molecular biology methods. Initially, the DNA extracted from the subjects of paternity tests was amplified by Single Specific Primer-Polymerase Chain Reaction (SSP-PCR) with a HLA-ReadyGene kit (Inno-Train Diagnostik GmbH, Kronberg, Germany), and immunofluorescence was detected in a 2% agarose gel according to the manufacturer's instructions. The gels were analyzed with Inno-Train SCORE software. At a later stage, when the newer technique of the SSP-PCR became available, the amplification of each DNA sample was performed with a HLA-FluoGene low resolution typing kit (Inno-Train Diagnostik GmbH, Kronberg, Germany) following the manufacturer's instructions. This set of results was evaluated with a FluoVista Analyzer (Inno-Train Diagnostik GmbH, Kronberg, Germany).

On the other hand, investigation of the HLA-A, HLA-B, HLA-C, and HLA-DRB1 genes from volunteers included in the Bone Marrow Donors National Program was performed by the Polymerase Chain Reaction Sequence-Specific Oligonucleotide (PCR-SSO) method, with a HISTO SPOT A, B, C, DRB1 kit (BAG Health Care GmbH, Germany). HLA data were analyzed with HISTO MATCH Software. Exons 2 and 3 for HLA-A, HLA-B, and HLA-C, as well as exon 2 for HLA-DRB1, were amplified in the process. HLA ambiguous typing was retested via SSP-PCR using the HLA A-B-DR SSP Combi Tray (CareDx, Stockholm, Sweden) and the HLA-C low resolution kit (CareDx, Stockholm, Sweden), according to the manufacturer's instructions. The results were processed with the Helmberg SCORE 5.00.41T software.

### 2.4. Statistical Analysis

The relative frequencies of the HLA-A, HLA-B, HLA-C, and HLA-DRB1 genes were expressed as ratios of their absolute frequencies (direct counting) to the total number of genes. Since the gametic phase was unknown, deviation from the Hardy-Weinberg equilibrium was appreciated with a test similar to Fisher's exact test performed locus by locus on an extended contingency table to arbitrary size [22, 23]. The average distance between two populations was computed as the distance between each pair of gene frequencies using the fixation index (*F*_ST_) formula provided by Rosenberg et al. [24]. We hypothesized that the *F*_ST_ distance is inversely proportional to the genetic relatedness of two populations. For each gene, we produced a relatedness hierarchy based on *F*_ST_ distance—top countries exhibiting the smallest average *F*_ST_ distance to the Transylvanian sample. A multiple correspondence analysis of the *F*_ST_ distances for the HLA-A, HLA-B, HLA-C, and HLA-DRB1 loci generated the overall relationships between the populations analyzed in this study.

We determined the 4-locus and 3-locus haplotypes present in our sample. However, only the first 50 most frequent 3-locus haplotypes (HLA-A, HLA-B, and HLA-DRB1) were used in the comparative analysis since they are more frequently reported in literature.

The frequencies published by Constantinescu et al. [12], Lebedeva [11], and Rendine et al. [16] were taken into consideration when calculating the sample size. In order to be able to detect low gene frequencies (0.01%), the sample size was increased by adding 532 subjects to the initial 2,262.

The chi-square test or Fisher's exact test was used to compare the allele frequencies between different samples when any values in the 2 × 2 expected tables were <5. *P* values were adjusted using the Bonferroni correction, considering the number of comparisons recorded.

For the statistical analysis, we used IBM SPSS 25.0 [25], Microsoft Excel (2016), and Arlequin 3.5.2.2 [22]. *P* values < 0.05 were considered significant.

## 3. Results

We identified 18 HLA-A different genes in 2,794 subjects, 30 HLA-B genes in 2,773 individuals, 13 HLA-C genes in 1,847 individuals, and 13 HLA-DRB1 genes in 2,719 individuals. Their frequencies are presented in Table 2. For all the considered HLA genes of the Transylvanian subjects, a statistically significant departure from the Hardy-Weinberg exact equilibrium test was observed (Table 2).

For 1,770 individuals, we obtained complete sets of data for all four HLA-A, HLA-B, HLA-C, and HLA-DRB1 loci. A total of 9,170 different haplotypes were identified in our sample. Based on the statistical analysis, the most frequent haplotypes were A^∗^01~B^∗^08~C^∗^07~DRB1^∗^03 (1.26%), A^∗^02~B^∗^18~C^∗^07~DRB1^∗^11 (0.77%), and A^∗^02~B^∗^08~C^∗^07~DRB1^∗^03 (0.41%).

2,832 different haplotypes were identified in the 2,708 individuals for whom we had obtained complete data for 3 loci only (HLA-A, HLA-B, and HLA-DRB1), 221 of which presented frequencies higher than 0.1%. The most frequent haplotypes were A^∗^01~B^∗^08~DRB1^∗^03 (1.70%), A^∗^02~B^∗^18~DRB1^∗^11 (1.45%), and A^∗^02~B^∗^35~DRB1^∗^11 (0.68%). About 11% of the total haplotype frequencies can be accounted for the 50 most frequent haplotypes. The 50 most common HLA-A~B~DRB1 haplotypes for the Transylvanian ethnic group (0.5%) are presented in Table 3.

In our sample, we found 400 (14.3%) individuals homozygous for the HLA-A locus, 219 (7.9%) for HLA-B, 284 (15.4%) for HLA-C, and 336 (12.4%) for the HLA-DRB1 loci. 651 (36.8%) of the 1770 subjects were homozygous: 12 of them (0.7%) for all four loci, 33 (1.9%) for three, 109 (6.2%) for two, and 497 (28.1%) for only one locus.

## 4. Discussion

The purpose of this study, to analyze HLA frequencies in the Transylvanian population and to compare them with European population groups of over 1,000 individuals, was achieved. In our pursuit, we considered being of interest to compare Transylvania with its neighboring countries and regions: Ukraine (north), Moldavia and the Republic of Moldova (east), Wallachia and Bulgaria (south), and Serbia and Hungary (west). Unfortunately, we did not find representative studies (*n* > 1,000) concerning the population of the Moldavia province, regarding the Republic of Moldova, Bulgaria, or Ukraine.

In regard to the most frequent genes, our results are similar with those reported in several other European population studies, in particular to those from neighbouring Serbia [14], Hungary [13], Wallachia [12], or Croatia [15].

HLA-A^∗^02, the most frequent HLA-A gene in the Transylvanian sample, was also the most frequent one in the Czech, Polish, Portuguese, Russian, and Italian populations, with no statistically significant differences being observed when their frequencies were compared (Table 4). However, the observed frequency of this locus was below the frequencies reported for Croatia, Serbia, Germany, or Hungary. HLA-A^∗^01 was the second most frequent gene in the analyzed sample, as well as in the population groups from Serbia, Czech Republic, Slovakia, Poland, Italy, Hungary, and Portugal. Its frequency was significantly different from those observed in Southern Europe populations such as the Greeks, Italians, and Portuguese, as well as in the Croatian and Wallachian ones.

Although the HLA-B genes analyzed in our study exhibited the highest degree of polymorphism, we managed to find several similarities with the results reported in other population studies in Europe (Table 5). HLA-B^∗^35, the most frequent HLA-B gene in the Transylvanian sample, was also found as the most frequent gene in the Wallachian, Greek, Serbian, Croatian, Slovakian, Polish, and Italian populations, its frequency being not statistically different to those observed in the Serbian, Croatian, Wallachian, and Portuguese ones (Table 5). Lower frequencies of the HLA-B^∗^35 gene were observed in Northern Europe.

The HLA-B^∗^18 gene was the second most frequent in our sample, as well as in the Wallachian group, its frequency being not statistically different to those observed in the Wallachian, Greek, Serbian, Italian, Hungarian, or Slovakian population groups. Concerning B^∗^07, B^∗^08, or B^∗^44, the Transylvanian group exhibited lower frequencies than those reported for Northern and Central Europe. However, they were higher than those observed in Italy or Greece. Although no statistically significant differences in terms of frequencies were observed in either case, these data suggest an increasing trend from Southeastern towards Northwestern Europe. A reverse pattern (in accordance with our findings) is observed for the HLA-B^∗^51 gene, more frequent in Southeastern than in Northwestern Europe. Of note, the frequencies we found for HLA-B^∗^50 and B^∗^53 displayed the lowest values when compared to all studied groups.

Regarding the HLA-C locus, the scarceness of relevant data (studies with *n* > 1,000) allowed statistical analyses with only four other European population groups (Table 6). Since HLA-C^∗^07 was the most frequent HLA-C gene in the Transylvanian sample, as in all four considered groups, one might conclude it is the most frequent HLA-C gene in the European populations (Table 6). The second most frequent gene was C^∗^04, as in the Czech, Polish, and French populations.

The most frequent HLA-DRB1 gene in the Transylvanian sample, DRB1^∗^11, was also the most frequent one in the Hungarian, Greek, Serbian, Croatian, Slovakian, and Italian populations, its frequency being not statistically different from the ones in the Serbian and Wallachian populations; while smaller than those observed in Italy and Greece, the DRB1^∗^11 frequency in our sample was higher than that in all other groups (Table 7).

As a conclusion of this univariate analysis, no statistically significant differences were found between the Transylvanian population and the Serbian one when considering 51 out of the 61 genes analyzed (a concordance of 83.6%). High concordance was also noted in comparisons with the Hungarian (50 genes—82.0%), Wallachian (48 genes—78.7%), and Croatian (47 genes—77.0%) populations (Tables 4–7).

**Figure 2:**
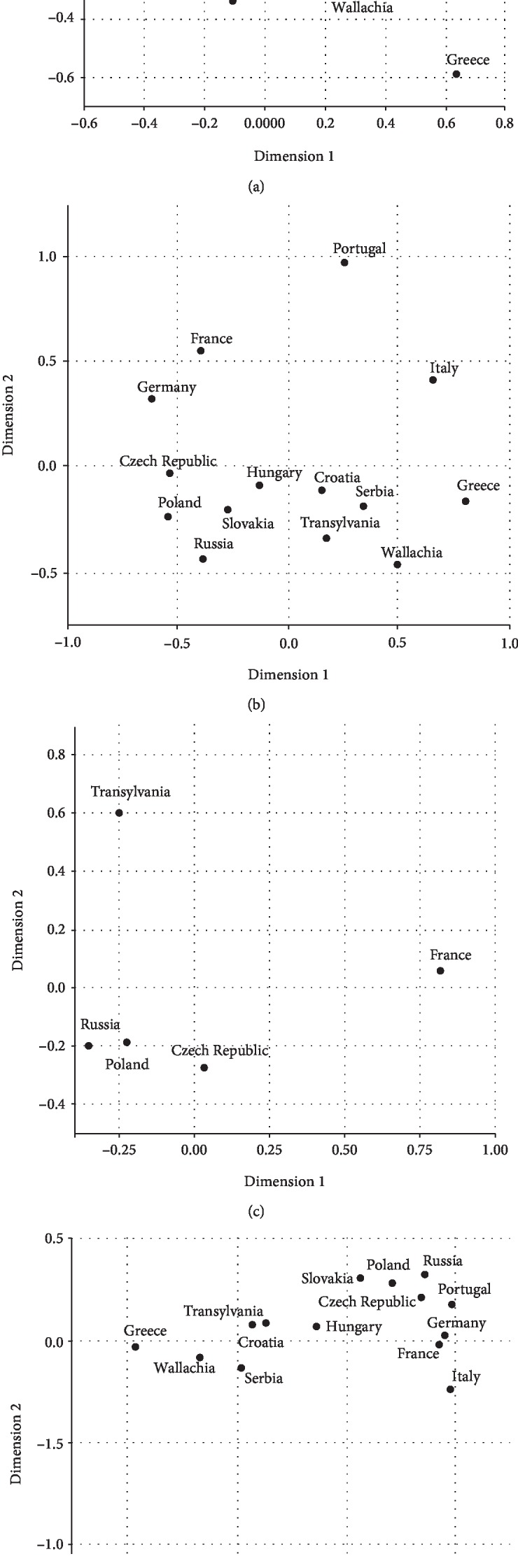


The multivariate analysis confirmed the results of the univariate analysis: Serbians, Hungarians, Wallachians, and Croatians were shown to be genetically closer to the Transylvanian population. Genetic *F*_ST_ distances between the gene frequencies of the Transylvanian sample and of all other population groups taken into consideration in this study using multiple correspondence analyses for the HLA-A, HLA-B, HLA-C, and HLA-DRB1 loci are shown in Figure 2. Our data are consistent with two studies in Hungary [13] and Serbia [14], both indicating that the Serbs were genetically the most related to the Romanians.

Our study was the first to determine the haplotypes in this population group, allowing possible comparisons with ancient genomes. Since most literature data refer to three-locus haplotypes (HLA-A~B~DRB1), only the 50 most frequent three-locus haplotypes are presented here (see Table 3).

Regarding the most common haplotypes, our results are similar to those reported for the neighboring populations. The most frequently observed haplotype, A^∗^01~B^∗^08~DRB1^∗^03, was in a similar position in several other European populations: Serbian [14], German [17], or Croatian [15]. The second most frequent in our sample, as well as in the Croatian [15] and Serbian [14] populations, the A^∗^02~B^∗^18~DRB1^∗^11 haplotype was reported to be the most common in two Bulgarian studies [18, 19] and fairly common in both Greek and Macedonian populations [20, 21]. In contrast, it was only ranked the 23^rd^ in a German population group [17]. Our third most frequent haplotype, A^∗^02~B^∗^35~DRB1^∗^11, was reported only in the 50^th^ place in the German population (0.3%) [17] and was not listed in the Bulgarian top 12 [19], in the Bulgarian top 16 [18], in the Serbian top 10 [14], or in the Croatian top 50 most frequent haplotypes [15]. However, this haplotype was reported as common in certain Southern Europe populations such as the Greeks and Lombardy Italians (1.4%) [11].

For an equiprobable population, the probability of an individual being homozygous in a 1,770 sample size is 4.8% for HLA-A, 2.8% for HLA-B, 7.14% for HLA-C, and 7.7% for HLA-DRB1. In our sample, we found 3 times more individuals homozygous for the HLA-A locus than expected, 2.8 times more individuals homozygous for the HLA-B locus, 2.2 times more homozygous individuals for the HLA-C locus, and 1.6 times more homozygous individuals for the HLA-DRB1 locus than in the equiprobable population sample. A *consanguineous* environment in *isolated mountain communities* might be accountable in this case [26, 27], a hypothesis supported by a statistically significant departure from the Hardy-Weinberg exact equilibrium found in our sample. Literature data revealed similarities for the HLA-A and HLA-B loci in a German population [28].

Another explanation for the statistically significant departure from the Hardy-Weinberg exact equilibrium could be the existence of two major ethnically distinct groups within the population: the Romanians (70.62%) and the Hungarians (17.92%). To confirm such an assumption, the two groups should be analyzed separately in future studies.

Although a sample size calculation was performed and a more than double number of individuals were enrolled in the study, the sample size was found to be too small for some rare genes (1 out of the 21 HLA-A genes, 5 of the 36 HLA-B genes, and 1 out of the 14 HLA-C genes). Some genes might not be present in the Transylvanian population at all (e.g., HLA-A^∗^80, which was not found in a considerably larger study on 159,311 Italians [16]). We recommend that further studies should consider larger sample sizes.

Another limitation of our study is that the population sample was not randomly selected from the general population, the selection process including only volunteer donors and paternity subjects consenting to participate in this study. However, taking into account the highly diverse origin of the Transylvanian sample, we consider that this aspect did not interfere significantly with our results.

## 5. Conclusions

This study provides information on a genetically imbalanced population subjected to intense migration in a continuously inhabited territory relatively isolated by a mountainous chain.

The most common genes found in the 2,794 analyzed individuals were HLA-A^∗^02, B^∗^35, C^∗^07, and DRB1^∗^11, while the most common haplotype in 1,770 individuals with complete data was A^∗^01~B^∗^08~C^∗^07~DRB1^∗^03.

Our findings are that genetically closest to the Transylvanian sample are the neighbouring populations from Serbia, Hungary, and Wallachia.

The data derived from this study can be considered an incipient database helpful for subsequent population and disease association studies or for donor recruitment planning at the regional level.

## Figures and Tables

**Table 1:** Resources which provided the frequencies of the HLA-A, HLA-B, HLA-C, and HLA-DRB1 genes in several European population groups.

Population group	Number of individuals sampled for the HLA-A, HLA-B, HLA-C, and HLA-DRB1 genes	Source	
		http://www.allelefrequencies.net [11], uploaded by	Published references
Wallachia	A = 5,252, B = 4,914, DRB1 = 5,072	—	Constantinescu et al. [12]
Hungary	A = 1,644; B = 1,653 DRB1 = 2,402	—	Inotai et al. [13]
Greece	A = 10,947, B = 11,061, *DRB*1 = 9,081	Iniotaki and Siorenta, 2012, Bone Marrow Registry	—
Serbia	A, B, DRB1 = 1,992	Andric et al., 2010, Bone Marrow Registry	Andric et al. [14]
Croatia	A, B, DRB1 = 4,000	Grubic et al., 2011, Bone Marrow Registry	Grubic et al. [15]
Czech Republic	A, B, DRB1 = 5,099, C = 4,669	Czech National Marrow Donors Registry, 2014	—
Slovakia	A = 3,693, B = 3,944, DRB1 = 3,744	Kušíková, 2013, Bone Marrow Registry	—
Poland	A, B, C, DRB1 = 2,907	Nowak et al., 2013, Bone Marrow Registry	—
Russia	A, B, C, DRB1 = 2,650	Lebedeva, 2009, Moscow Stem Cell Bank	—
Germany	A, B, DRB1 = 11,407	Seidl et al., 2005, Bone Marrow Registry	—
France	A, B, C, DRB1 = 6,094	Alizadeh et al., 2006, Bone Marrow Registry	—
Italy	A, B = 159,311, DRB1 = 40,071	Rendine et al., 1998, Bone Marrow Registry	Rendine et al. [16]
Portugal	A, B, DRB1 = 17,420	Ligeiro, 2009, Bone Marrow Registry	—
Spain	DRB1 = 1,818	Garcia-Sanz, 2007, Bone Marrow Registry	—

**Table 2:** The HLA-A (*n* = 2,794), HLA-B (*n* = 2,773), HLA-C (*n* = 1,847), and HLA-DRB1 (*n* = 2,719) gene frequencies in the Transylvanian sample.

Gene HLA-A	Frequency (%) *n* = 2,794 *P* value = 0.005	Gene HLA-B	Frequency (%) *n* = 2,773 *P* value < 0.001	Gene HLA-C	Frequency (%) *n* = 1,847 *P* value < 0.001	Gene HLA-DRB1	Frequency (%) *n* = 2,719 *P* value < 0.001
A^∗^01	14.2	B^∗^07	5.7	C^∗^01	5.4	DRB1^∗^01	9.8
A^∗^02	26.9	B^∗^08	8.6	C^∗^02	7.2	DRB1^∗^03	11.4
A^∗^03	11.1	B^∗^13	3.8	C^∗^03	7.7	DRB1^∗^04	8.8
A^∗^11	7.3	B^∗^14	2.5	C^∗^04	15.1	DRB1^∗^07	10.6
A^∗^23	2.7	B^∗^15	3.9	C^∗^05	3.9	DRB1^∗^08	2.5
A^∗^24	11.8	B^∗^18	11.2	C^∗^06	8.9	DRB1^∗^09	0.2
A^∗^25	3.3	B^∗^27	5	C^∗^07	24.8	DRB1^∗^10	1.1
A^∗^26	5.1	B^∗^35	13.9	C^∗^08	2.7	DRB1^∗^11	19
A^∗^29	1.6	B^∗^37	1	C^∗^12	13.9	DRB1^∗^12	1.7
A^∗^30	2.6	B^∗^38	4.3	C^∗^14	2.4	DRB1^∗^13	11.4
A^∗^31	2.3	B^∗^39	2.3	C^∗^15	5.2	DRB1^∗^14	4.3
A^∗^32	4.5	B^∗^40	5.1	C^∗^16	1.7	DRB1^∗^15	8.8
A^∗^33	2.1	B^∗^41	1.6	C^∗^17	1.2	DRB1^∗^16	10.3
A^∗^34	0.04	B^∗^42	0.02	C^∗^18	0	—	—
A^∗^36	0	B^∗^44	10	—	—	—	—
A^∗^43	0	B^∗^45	0.3	—	—	—	—
A^∗^66	0.9	B^∗^46	0.1	—	—	—	—
A^∗^68	3.1	B^∗^47	0.6	—	—	—	—
A^∗^69	0.2	B^∗^48	0.3	—	—	—	—
A^∗^74	0.2	B^∗^49	1.7	—	—	—	—
A^∗^80	0	B^∗^50	1	—	—	—	—
—	—	B^∗^51	8.9	—	—	—	—
—	—	B^∗^52	2.2	—	—	—	—
—	—	B^∗^53	0.2	—	—	—	—
—	—	B^∗^54	0.02	—	—	—	—
—	—	B^∗^55	1.8	—	—	—	—
—	—	B^∗^56	0.8	—	—	—	—
—	—	B^∗^57	2	—	—	—	—
—	—	B^∗^58	1.3	—	—	—	—
—	—	B^∗^59	0	—	—	—	—
—	—	B^∗^67	0	—	—	—	—
—	—	B^∗^73	0.04	—	—	—	—
—	—	B^∗^78	0	—	—	—	—
—	—	B^∗^81	0	—	—	—	—
—	—	B^∗^82	0	—	—	—	—
—	—	B^∗^83	0	—	—	—	—

*n*: number of analyzed subjects; *P*: value of the Hardy-Weinberg exact equilibrium test for gene frequencies at each locus.

**Table 3:** The most frequent 50 HLA-A~B~DRB1 haplotypes from the 2,708 analyzed individuals presented in descending order of their frequency.

Rank	HLA-A~B~DRB1 haplotype	Haplotype frequency (%) *n* = 2,708	Rank	HLA-A~B~DRB1 haplotype	Haplotype frequency (%) *n* = 2,708
1	A^∗^01~B^∗^08~DRB1^∗^03	1.70	26	A^∗^02~B^∗^35~DRB1^∗^13	0.33
2	A^∗^02~B^∗^18~DRB1^∗^11	1.45	27	A^∗^02~B^∗^51~DRB1^∗^13	0.33
3	A^∗^02~B^∗^35~DRB1^∗^11	0.68	28	A^∗^03~B^∗^35~DRB1^∗^11	0.32
4	A^∗^02~B^∗^08~DRB1^∗^03	0.66	29	A^∗^02~B^∗^27~DRB1^∗^01	0.32
5	A^∗^02~B^∗^44~DRB1^∗^11	0.66	30	A^∗^01~B^∗^18~DRB1^∗^11	0.31
6	A^∗^24~B^∗^35~DRB1^∗^11	0.60	31	A^∗^02~B^∗^07~DRB1^∗^11	0.31
7	A^∗^03~B^∗^35~DRB1^∗^01	0.55	32	A^∗^11~B^∗^18~DRB1^∗^11	0.31
8	A^∗^24~B^∗^18~DRB1^∗^11	0.55	33	A^∗^02~B^∗^38~DRB1^∗^13	0.30
9	A^∗^02~B^∗^51~DRB1^∗^11	0.54	34	A^∗^25~B^∗^18~DRB1^∗^15	0.29
10	A^∗^02~B^∗^51~DRB1^∗^16	0.53	35	A^∗^01~B^∗^35~DRB1^∗^11	0.29
11	A^∗^02~B^∗^44~DRB1^∗^16	0.53	36	A^∗^02~B^∗^18~DRB1^∗^07	0.29
12	A^∗^02~B^∗^35~DRB1^∗^01	0.49	37	A^∗^23~B^∗^44~DRB1^∗^07	0.29
13	A^∗^11~B^∗^35~DRB1^∗^01	0.44	38	A^∗^02~B^∗^35~DRB1^∗^16	0.28
14	A^∗^02~B^∗^18~DRB1^∗^16	0.43	39	A^∗^02~B^∗^40~DRB1^∗^13	0.28
15	A^∗^02~B^∗^13~DRB1^∗^07	0.42	40	A^∗^24~B^∗^44~DRB1^∗^11	0.27
16	A^∗^03~B^∗^18~DRB1^∗^11	0.42	41	A^∗^02~B^∗^18~DRB1^∗^15	0.26
17	A^∗^03~B^∗^07~DRB1^∗^15	0.42	42	A^∗^02~B^∗^44~DRB1^∗^01	0.26
18	A^∗^24~B^∗^08~DRB1^∗^03	0.42	43	A^∗^03~B^∗^08~DRB1^∗^03	0.26
19	A^∗^01~B^∗^08~DRB1^∗^11	0.40	44	A^∗^03~B^∗^51~DRB1^∗^11	0.26
20	A^∗^02~B^∗^44~DRB1^∗^07	0.39	45	A^∗^02~B^∗^15~DRB1^∗^04	0.26
21	A^∗^02~B^∗^44~DRB1^∗^04	0.37	46	A^∗^32~B^∗^35~DRB1^∗^11	0.26
22	A^∗^02~B^∗^44~DRB1^∗^13	0.36	47	A^∗^01~B^∗^08~DRB1^∗^13	0.25
23	A^∗^02~B^∗^07~DRB1^∗^15	0.35	48	A^∗^01~B^∗^08~DRB1^∗^16	0.25
24	A^∗^02~B^∗^27~DRB1^∗^16	0.34	49	A^∗^02~B^∗^40~DRB1^∗^11	0.25
25	A^∗^11~B^∗^35~DRB1^∗^11	0.33	50	A^∗^01~B^∗^40~DRB1^∗^14	0.25

**Table 4:** Comparison of HLA-A gene frequencies in the Transylvanian (*n* = 2,794) vs. other European population groups.

Gene	Greece	Serbia	Croatia	Czech Republic	Slovakia	Poland	Russia	Germany	France	Italy	Portugal	Hungary	Wallachia
A^∗^01		X	X	X	X	X		X	X	X		X	
A^∗^02				X		X	X			X	X	X	
A^∗^03		X	X		X					X	X	X	X
A^∗^11	X	X	X		X	X	X		X		X		X
A^∗^23	X	X	X	X	X	X	X	X	X	X		X	X
A^∗^24		X	X		X					X	X		X
A^∗^25		X	X	X								X	X
A^∗^26	X	X	X	X	X	X	X			X		X	X
A^∗^29	X			X	X	X	X					X	X
A^∗^30		X		X	X	X	X	X	X		X	X	X
A^∗^31	X	X	X	X	X	X	X	X	X	X	X	X	X
A^∗^32		X	X		X			X	X	X	X	X	X
A^∗^33	X	X	X				X			X		X	X
A^∗^34	X	X	X	X	X	X	X	X	X	X		X	X
A^∗^66	X			X	X	X	X				X	X	
A^∗^68	X	X		X	X	X	X	X	X	X		X	X
A^∗^69		X	X	X	X	X	X	X	X		X	X	X
A^∗^74	X			X		X		X	X		X	X	X
*n*	10,947	1,992	4,000	5,099	3,693	2,907	2,650	11,407	6,094	159,311	17,420	1,644	5252

*n*: number of analyzed subjects; X: not statistically significant *P* value. Resources presented in Table 1 provided the gene frequency references.

**Table 5:** Comparison of HLA-B gene frequencies in the Transylvanian (*n* = 2,773) vs. other European population groups.

Gene	Greece	Serbia	Croatia	Czech Republic	Slovakia	Poland	Russia	Germany	France	Italy	Portugal	Hungary	Wallachia
B^∗^07		X								X	X		X
B^∗^08		X	X		X	X	X		X				
B^∗^13	X	X	X		X			X		X		X	X
B^∗^14	X	X	X	X	X	X	X	X				X	X
B^∗^15	X	X	X									X	X
B^∗^18	X	X								X		X	X
B^∗^27		X	X	X	X	X	X	X	X			X	X
B^∗^35		X	X								X		X
B^∗^37	X	X	X	X	X	X	X	X	X	X	X	X	X
B^∗^38		X	X	X	X		X			X		X	
B^∗^39	X	X	X	X	X	X	X	X	X	X		X	X
B^∗^40			X	X	X	X	X		X			X	X
B^∗^41	X	X		X	X	X	X			X		X	X
B^∗^42	X	X	X	X	X	X	X	X	X	X	X	X	X
B^∗^44		X	X	X	X	X	X			X			X
B^∗^45	X	X	X	X	X	X	X	X	X	X		X	X
B^∗^46	X	X		X	X	X	X	X		X		X	X
B^∗^47	X	X		X	X	X	X	X	X	X	X	X	X
B^∗^48		X	X	X	X	X	X				X	X	X
B^∗^49			X	X	X	X	X	X	X			X	X
B^∗^50	X	X	X	X		X	X	X	X			X	
B^∗^51									X		X		X
B^∗^52	X		X		X	X	X					X	
B^∗^53		X		X	X	X	X	X				X	
B^∗^54	X	X	X	X	X	X	X	X	X	X	X	X	X
B^∗^55		X	X	X	X				X			X	X
B^∗^56	X	X	X	X	X		X	X	X		X	X	X
B^∗^57	X	X	X		X				X		X	X	
B^∗^58	X	X	X	X			X	X	X			X	X
B^∗^73		X	X	X	X	X	X	X	X		X	X	X
*n*	11,061	1,992	4,000	5,099	3,944	2,907	2,650	11,407	6,094	159,311	17,420	1,653	4,914

*n*: number of analyzed subjects; X: not statistically significant *P* value. Resources presented in Table 1 provided the gene frequency references.

**Table 6:** Comparison of HLA-C gene frequencies in the Transylvanian (*n* = 1,847) vs. other European population groups.

Gene	Czech Republic	Poland	Russia	France
C^∗^01		X	X	
C^∗^02		X	X	
C^∗^03			X	
C^∗^04				
C^∗^05	X	X	X	
C^∗^06				
C^∗^07		X	X	
C^∗^08	X	X	X	
C^∗^12		X	X	
C^∗^14			X	X
C^∗^15				
C^∗^16		X	X	
C^∗^17	X	X		
*n*	4,669	2,907	2,650	6,094

*n*: number of analyzed subjects; X: not statistically significant *P* value. Resources presented in Table 1 provided the gene frequency references.

**Table 7:** Comparison of HLA-DRB1 gene frequencies in the Transylvanian (*n* = 2,719) vs. other European population groups.

Gene	Greece	Serbia	Croatia	Czech Republic	Slovakia	Poland	Russia	Germany	France	Italy	Portugal	Spain	Hungary	Wallachia
DRB1^∗^01		X	X	X	X	X	X	X	X	X		X	X	X
DRB1^∗^03		X	X	X	X	X		X	X	X	X	X	X	X
DRB1^∗^04	X	X	X			X				X				X
DRB1^∗^07			X										X	
DRB1^∗^08		X			X					X		X	X	
DRB1^∗^09	X	X	X										X	
DRB1^∗^10		X	X	X	X	X	X	X	X	X	X	X	X	X
DRB1^∗^11		X												X
DRB1^∗^12	X	X	X	X		X		X	X		X	X	X	X
DRB1^∗^13			X		X	X							X	X
DRB1^∗^14		X	X		X				X				X	
DRB1^∗^15	X	X	X								X	X		X
DRB1^∗^16		X	X											X
*n*	9,081	1,992	4,000	5,099	3,744	2,907	2,650	11,407	6,094	40,071	17,420	1,818	2,402	5,072

*n*: number of analyzed subjects; X: not statistically significant *P* value. Resources presented in Table 1 provided the gene frequency references.

## Data Availability

All relevant data is within the paper. All raw data remains in the possession of the authors of the article.
